# Diagnostic and prognostic value of serum markers CEA, CA50, CA19-9, CA72-4, and CYFRA21-1 in colon cancer

**DOI:** 10.5937/jomb0-55648

**Published:** 2026-01-06

**Authors:** Ze Song, Jun Tang, Min Wu, Zhi Li, Yingqiang Zhang

**Affiliations:** 1 The Seventh Affiliated Hospital, Sun Yat-sen University, Department of Oncology, Shenzhen, Guangdong Province, China; 2 Southern University of Science and Technology, Department of Vascular and Interventional Oncology, Shenzhen, Guangdong Province, China; 3 Huizhou Municipal Centre Hospital, Office of GCP, Department of Pharmacy, Huizhou City, Guangdong Province, China; 4 Hunan Provincial People's Hospital, the First Affiliated Hospital of Hunan Normal University, Department of Radiology, Changsha city, Hunan province, China; 5 The Seventh Affiliated Hospital, Sun Yat-sen University, Department of Interventional Radiology, Shenzhen, Guangdong Province, China

**Keywords:** serum tumour markers, colon cancer, CEA, CA19-9, CYFRA21-1, early screening, prognosis, recurrence and metastasis, serumski tumour markeri, karcinom debelog creva, CEA, CA19-9, CYFRA21-1, rano skrining testiranje, prognoza, recidiv i metastaze

## Abstract

**Background:**

This study aimed to assess the clinical utility of serum tumour markers - carcinoembryonic antigen (CEA), carbohydrate antigen (CA) 50, CA19-9, CA72-4, and the cytokeratin 19 fragment (CYFRA21-1) - for early detection and prognostic evaluation in colon cancer.

**Methods:**

A total of 50 patients diagnosed with colon cancer and 50 healthy individuals were enrolled. Serum levels of CEA, CA50, CA19-9, CA72-4, and CYFRA21-1 were quantified and compared between groups. Correlations with tumour staging, recurrence, and metastasis were also analysed.

**Results:**

All five serum markers were significantly elevated in colon cancer patients compared to controls (P&lt; 0.05). Marker levels were notably higher in advanced-stage cases (stages III-IV) than in early-stage disease (stages I-II) (P&lt; 0.05). Following surgery, marker levels declined significantly (P&lt; 0.05), except in patients with recurrence or metastasis, who showed elevated preoperative values (P&lt; 0.05). Combined detection yielded significantly higher positive detection rates, sensitivity, and specificity than individual marker assessments (P&lt; 0.05).

**Conclusions:**

CEA, CA50, CA19-9, CA72-4, and CYFRA21-1 are clinically relevant markers for the detection, staging, and prognostic prediction of colon cancer. Combined serum marker analysis substantially improves diagnostic precision and supports early screening and outcome assessment.

## Abstract (sr)

**Uvod:**

Cilj ove studije bio je da se proceni klinička korisnost serumskih tumourskih markera - karcinoembrionalnog antigena (CEA), karbohidratnog antigena (CA) 50, CA19-9, CA72-4 i fragmenta citokeratina 19 (CYFRA21-1) - za ranu detekciju i prognostičku procenu kod karcinoma debelog creva.

**Metode:**

U studiju je bilo uključeno ukupno 50 pacijenata sa dijagnozom karcinoma debelog creva i 50 zdravih ispitanika. Kvantifikovani su nivoi CEA, CA50, CA19-9, CA72-4 i CYFRA21-1 u serumu i upoređeni između grupa. Takođe su analizirane korelacije sa stadijumom tumoura, recidivom i metastazama.

**Rezultati:**

Kod pacijenata sa karcinomom debelog creva svi markeri su bili značajno poviseni u odnosu na kontrolnu grupu (P&lt; 0,05). Nivoi markera su bili znatno visi u uznapredovalim stadijumima bolesti (stadijumi III-IV) u poređenju sa ranim stadijumima (stadijumi I-II) (P&lt; 0,05). Nakon operacije, zabeležen je značajan pad nivoa markera (P&lt; 0,05), osim kod pacijenata sa recidivom ili metastazama, kod kojih su preoperativne vrednosti bile povisene (P&lt; 0,05). Kombinovano određivanje markera pokazalo je značajno visu stopu pozitivne detekcije, senzitivnost i specifičnost u poređenju sa pojedinačnim testiranjima (P&lt; 0,05).

**Zaključak:**

CEA, CA50, CA19-9, CA72-4 i CYFRA21-1 su klinički značajni markeri za detekciju, određivanje stadijuma i prognostičku procenu karcinoma debelog creva. Kombinovana analiza serumskih markera značajno poboljšava dijagnostičku preciznost i potpomaže ranu detekciju i procenu ishoda.

## Introduction

Colon cancer has emerged as a major public health concern, ranking just behind gastric and oesophagal cancers in prevalence in China [1] [2] [3]. In recent years, shifts in lifestyle and dietary patterns have contributed to a continuous rise in its incidence, significantly impairing the quality of life for affected individuals. Due to its insidious onset and lack of symptoms in early stages, colon cancer is frequently diagnosed late, resulting in missed opportunities for optimal treatment and, subsequently, reduced therapeutic efficacy [4] [5] [6] [7]. While radical surgery remains among the most effective curative approaches, the rate of postoperative local recurrence and distant metastasis is approximately 65%, making it a principal contributor to the high mortality rate associated with the disease [8] [9] [10]. Therefore, the development of reliable diagnostic strategies and markers for early detection is essential to improve disease management and patient survival outcomes.

Traditionally, diagnostic evaluation of colon cancer has relied on imaging techniques such as ultrasound and computed tomography (CT). However, these modalities are limited by their complexity, equipment demands, and high associated costs, which impose a substantial financial burden on healthcare systems and patients [11] [12] [13]. Furthermore, imaging modalities are often less sensitive in detecting early-stage tumours or minimal residual disease, contributing to diagnostic delays. As such, there is a pressing need for diagnostic tools that are simple, cost-effective, and accurate.

In this context, serum tumour markers - biochemical substances produced or released during tumourigenesis - have gained attention as promising tools for the detection and monitoring of malignancies. These markers can be measured noninvasively and repeatedly, making them suitable for both initial screening and longitudinal follow-up [14] [15] [16]. Among the markers investigated in gastrointestinal oncology, carcinoembryonic antigen (CEA), glycoprotein antigen CA50, CA19-9, CA72-4, and the soluble fragment of cytokeratin 19 (CYFRA21-1) are of particular interest for colon cancer diagnosis [17] [18] [19]. Individually, these markers have demonstrated varying degrees of sensitivity and specificity, yet their clinical utility remains limited when used in isolation. While CEA is widely applied in gastrointestinal malignancies, especially colorectal cancer, its prognostic and predictive value in recurrence and metastasis is still debated due to inconsistent findings across studies.

To address these limitations, recent research has explored the value of combined detection strategies, where multiple markers are assessed simultaneously to improve diagnostic accuracy. However, data on the comparative performance of such combined panels in the context of colon cancer, particularly in relation to staging, recurrence, and postoperative monitoring, remain insufficient. This study was designed to evaluate the clinical utility of five serum tumour markers - CEA, CA50, CA19-9, CA72-4, and CYFRA21-1 - in the early detection, staging, and prognostic assessment of colon cancer, with the aim of identifying a cost-effective and clinically applicable diagnostic approach.

## Materials and methods

### Study design and participants

This study included 50 patients diagnosed with colon cancer who were treated at The Seventh Affiliated Hospital, Sun Yat-sen University, Shenzhen 518107, Guangdong Province, China, between August 2023 and August 2024. The case group consisted of 27 males and 23 females, with a mean age of 63.27±11.34 years. All participants in the case group were diagnosed based on both clinical and pathological criteria. Tumour staging, according to the tumour-node-metastasis (TNM) classification, revealed 13 cases at stage I, 9 at stage II, 12 at stage III, and 16 at stage IV

A control group of 50 healthy individuals undergoing routine physical examinations was also enrolled, comprising 28 males and 22 females with a mean age of 64.28±12.73 years. There were no statistically significant differences in age or gender between the two groups (P>0.05), indicating baseline comparability.

The study protocol was approved by the Medical Ethics Committee of The Seventh Affiliated Hospital, Sun Yat-sen University, Shenzhen 518107, Guangdong Province, China. Informed consent was obtained from all patients and their families after providing detailed information about the study.

### Inclusion criteria

A. Age >18 years

B. Histologically and clinically confirmed diagnosis of colon cancer

C. No history of major organ dysfunction (digestive, cardiac, hepatic, renal)

D. Pathological TNM stages I to III, based on the National Comprehensive Cancer Network (NCCN) 2013 guidelines

### Exclusion criteria

A. Presence of malignancies at sites other than the colon

B. Inability to comply with treatment, follow-up, or re-evaluation protocols

### Sample collection and tumour marker analysis

Fasting venous blood samples (5 mL) were collected from control subjects during their routine physical examinations and from patients in the case group at multiple time points: upon admission, preoperatively, postoperatively, and one year after surgery. The samples were centrifuged to isolate serum, which was stored at -20°C until analysis.

Serum levels of CEA, CA50, CA19-9, CA72-4, and CYFRA21-1 were measured using the ES-300 enzyme immunoassay analyser (Boehringer, Germany). Commercial enzyme immunoassay kits for all five markers were obtained from Invitrogen (USA).

### Reference ranges for tumour markers

The upper limits of normal values for each tumour marker were defined as follows:

- CEA: <5 ng/mL
- CA50: <25 IU/mL
- CA19-9: <37 IU/mL
- CA72-4: <6.9 IU/mL
- CYFRA21-1: <3.3 ng/mL

Any value exceeding these thresholds was classified as positive. For the purpose of combined detection, a result was considered positive if any one of the five markers exceeded its reference range. Sensitivity, specificity, and positive detection rates were calculated for both individual and combined marker assessments.

### Statistical analysis

All statistical analyses were conducted using SPSS version 20.0. Continuous variables that conformed to normal distribution were expressed as mean ± standard deviation (x̄ ± s) and compared between groups using the independent samples t-test. Categorical data were presented as percentages and analysed using the chi-square (χ^2^) test. All experiments were repeated three times for reliability. A significance level of =0.05 was used, with P<0.05 considered statistically significant.

## Results

### Patient demographics

The study population was comprised of 100 individuals, divided equally into two groups: 50 patients diagnosed with colon cancer (case group) and 50 healthy participants (control group). The case group included 27 males and 23 females with a mean age of 63.27±11.34 years. The control group consisted of 28 males and 22 females, with a mean age of 64.28±12.73 years. No statistically significant differences were observed in gender or age distribution between the groups (P>0.05), confirming their baseline comparability.

Within the case group, TNM staging revealed 13 patients at stage I, 9 at stage II, 12 at stage III, and 16 at stage IV. Based on this, 28 patients were categorised as having early-stage disease (stages I-II), and 22 were considered late-stage (stages III-IV).

### Comparison of tumour marker levels between groups

**Figure 1:**
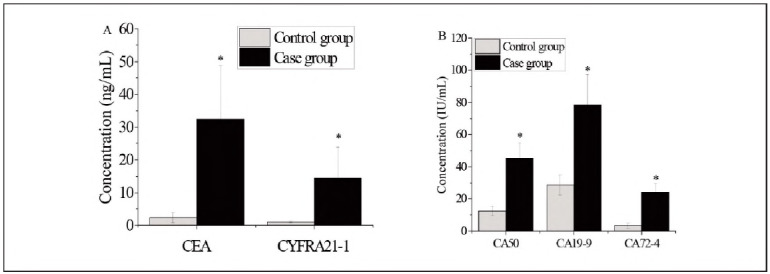
Tumour marker expression in the case and control groups. A. CEA and CYFRA21-1; B. CA50, CA19-9, and CA72-4. *Note: Differences between groups were statistically significant (P < 0.05).

Serum levels of CEA, CA50, CA19-9, CA72-4, and CYFRA21-1 were significantly elevated in the colon cancer group compared to the control group (P<0.05), as illustrated in Figure 1.

### Tumour marker expression by cancer stage

**Figure 2:**
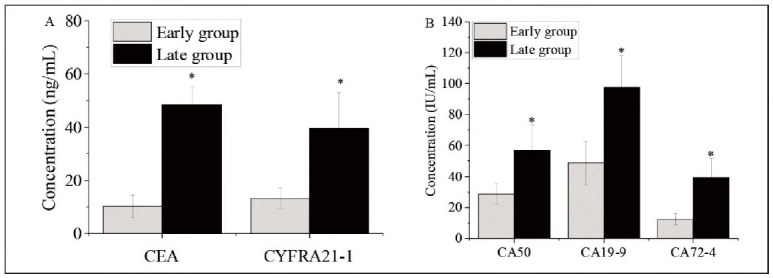
Tumour marker expression in early vs. late-stage colon cancer. A. CEA and CYFRA21-1; B. CA50, CA19-9, and CA72-4. *Note: Intergroup differences were statistically significant (P < 0.05).

Among patients with colon cancer, those in the late-stage group (stages III-IV) demonstrated significantly higher serum levels of all five tumour markers compared to those in the early-stage group (stages I-II) (P<0.05), as depicted in Figure 2.

### Tumour marker changes in recurrent and metastatic disease

**Figure 3:**
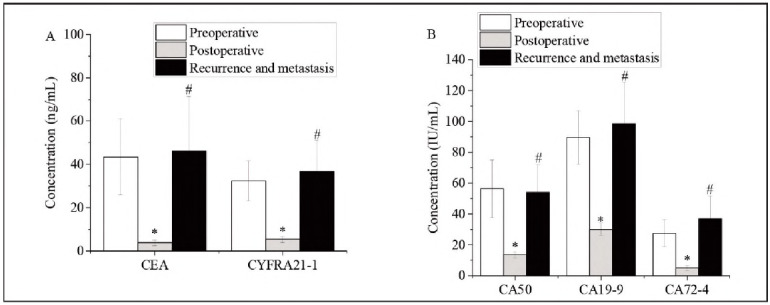
Tumour marker levels before and after recurrence/metastasis. A. CEA and CYFRA21-1; B. CA50, CA19-9, and CA72-4. *Note: Differences before and after recurrence/metastasis were significant (P < 0.05, #P<0.05).

All 50 patients in the case group underwent surgical treatment. Postoperative levels of CEA, CA50, CA19-9, CA72-4, and CYFRA21-1 were significantly decreased compared to preoperative levels (P<0.05). Among 23 patients who experienced postoperative recurrence or metastasis, preoperative marker levels were significantly higher than their postoperative values (P<0.05). However, no statistically significant differences were found between pre- and postoperative levels in the recurrence/metastasis subgroup (P>0.05), as shown in Figure 3.

### Diagnostic performance: Single vs. combined marker detection

Among the individual markers, CEA yielded the highest positive detection rate at 42.0%. Combined detection of the five markers produced a significantly higher positive rate (86.0%) compared to any single marker (P<0.05). However, the difference between combined detection and individual marker results was not statistically significant for certain markers (P>0.05). Data are summarised in Table 1.

**Table 1:** Diagnostic comparison of single vs. combined marker detection in colon cancer. *Note: Combined detection showed a statistically significant improvement in diagnostic performance (P<0.05).

Detection<br>methods	Positive rate<br>[n (%)]	Sensitivity<br>(%)	Specificity<br>(%)
CEA	21 (42.00	76.9	70.2
CA50	22 (44.0)	73.3	66.6
CA19-9	16 (32.0)	75.0	71.7
CA72-4	18 (36.0)	85.7	83.3
CYFRA21-1	19 (38.0)	88.8	73.1
The combined<br>detection	43 (86.0) *	95.1	55.5

### Detection of recurrence and metastasis: single vs. combined detection

For diagnosing recurrence and metastasis, combined detection again outperformed individual markers, showing higher positive rates and sensitivity. These improvements were statistically significant compared to single-marker tests (P<0.05 for positive rate; P>0.05 for some sensitivity comparisons). Results are presented in Table 2.

**Table 2:** Diagnostic comparison of single vs. combined tumour marker detection in recurrent/metastatic colon cancer. *Note: Combined detection showed statistically significant advantages over single-marker methods (P<0.05).

Detection<br>methods	Positive rate<br>[n (%)]	Sensitivity<br>(%)	Specificity<br>(%)
CEA	10 (43.4)	72.7	83.3
CA50	9 (39.1)	54.5	75.0
CA19-9	8 (34.7)	44.4	71.4
CA72-4	11 (47.8)	63.6	66.6
CYFRA21-1	13 (56.5)	72.7	58.3
The combined<br>detection	21 (91.3) *	95.0	33.3

## Discussion

The global incidence of colon cancer continues to rise, with an estimated annual increase of approximately 2.3%. In developed nations, it is currently the second leading cause of cancer-related mortality, while in China, it ranks fifth among malignant tumours [20] [21] [22]. Currently, the cornerstone of colon cancer treatment is surgical resection in combination with chemoradiotherapy. Reported five-year survival rates following surgery are approximately 50%, underscoring the critical importance of early detection, diagnosis, and intervention to improve patient outcomes [23] [24] [25]. In recent years, advances in molecular biology have facilitated the application of tumour markers for both preoperative screening and prognostic assessment. Compared to imaging or invasive diagnostic procedures, these serological methods offer advantages in terms of patient compliance and diagnostic efficiency [26] [27] [28] [29]. Commonly investigated markers include carcinoembryonic antigen (CEA), CA50, CA19-9, CA72-4, and CYFRA21-1.

CEA is a monomeric glycoprotein classified as an embryonic oncogenic antigen, primarily present in embryonic serum and gastrointestinal tissues. It exhibits minimal expression in normal adult tissues but is significantly upregulated in malignancies such as pancreatic, oesophagal, and gastrointestinal cancers [30]. Its diagnostic sensitivity in gastrointestinal malignancies is approximately 60%, and it demonstrates moderate to high sensitivity in colon cancer specifically [31]. Consistent with previous findings, our study revealed significantly elevated CEA levels in colon cancer patients compared to healthy controls (P<0.05), with a corresponding positive rate of 42.0%, sensitivity of 76.9%, and specificity of 70.2%.

CA50, a sialoglycoprotein with limited expression in normal tissues, is frequently elevated in liver, lung, gastric, colon, and biliary tract cancers. Literature suggests that up to 75% of colon cancer patients exhibit increased CA50 levels [32], which aligns with our findings. In the present study, CA50 demonstrated a positive rate of 44.0%, a sensitivity of 73.3%, and a specificity of 66.6%.

CA19-9, an oligosaccharide antigen secreted by adenocarcinoma cells, serves as a useful biomarker for various gastrointestinal tumours, including gastric, pancreatic, and colon cancers [33]. Our data showed significantly higher CA19-9 levels in the case group than in the control group (P<0.05), with a positive rate of 32.0%, sensitivity of 75.0%, and specificity of 71.7%.

CA72-4, a mucin-like glycoprotein, is predominantly expressed in gastrointestinal and pancreatic cancers and is widely used for malignancy screening. Our results confirmed that CA72-4 levels were significantly elevated in patients with colon cancer compared to healthy individuals, with a positive rate of 36.0%, sensitivity of 85.7%, and specificity of 83.3%. These findings are consistent with those of Wang et al. [34], who reported that CA72-4 demonstrated superior sensitivity compared to both CEA and CA19-9 in diagnosing colon and gastric cancers.

CYFRA21-1, a soluble fragment of cytokeratin 19, was also significantly elevated in the case group. It achieved a positive rate of 38.0%, sensitivity of 88.8%, and specificity of 73.1%, reinforcing its diagnostic utility.

Furthermore, the combined detection of all five markers yielded a significantly higher positive detection rate than any individual marker (P<0.05). Although differences between specific single-marker and combined detection results did not reach statistical significance (P>0.05), the overall enhancement in diagnostic sensitivity and reliability supports the use of multiplex detection strategies. This emphasises the clinical relevance of CEA, CA50, CA19-9, CA72-4, and CYFRA21-1 for early diagnosis and malignancy assessment in colon cancer. While individual markers demonstrated limited sensitivity when used in isolation, their combined application markedly improved detection rates.

Prognostic evaluation in colon cancer traditionally relies on pathological staging, which remains a gold standard. However, inaccurate staging can negatively impact treatment planning and prognosis. Accumulating clinical evidence supports the use of tumour markers in monitoring therapeutic efficacy and assessing disease progression [35]. Our results demonstrated that the expression levels of all five markers were significantly higher in late-stage disease compared to early-stage (P<0.05), indicating a correlation between marker elevation and tumour progression.

In addition to diagnostic value, tumour markers also serve as valuable tools for evaluating surgical outcomes and recurrence risk. In this study, patients were followed for one year postoperatively, and their marker levels were assessed preoperatively, postoperatively, and upon recurrence or metastasis. Post-treatment, levels of all markers showed significant reductions, while patients who developed recurrence or metastasis exhibited elevated marker levels. These findings underscore the potential value of integrating multi-marker serum detection into routine diagnostic protocols for colon cancer. The use of CEA, CA50, CA19-9, CA72-4, and CYFRA21-1 as a combined panel may enhance early-stage detection, especially in asymptomatic individuals or those at elevated risk. Additionally, monitoring these markers during followup could facilitate the earlier identification of recurrence or metastasis, thereby informing more timely therapeutic interventions. Incorporating this biomarker strategy into existing clinical workflows may contribute to improved diagnostic accuracy and better long-term patient management.

However, several limitations of this study should be acknowledged. First, it was conducted at a single centre, which may restrict the generalizability of the results to other populations or healthcare settings. Second, the follow-up period was limited to one year, preventing a comprehensive evaluation of the longterm prognostic value of these markers. Third, this study did not incorporate direct comparisons with other diagnostic modalities such as colonoscopy, faecal occult blood tests, or circulating tumour DNA (ctDNA), which are increasingly used in clinical practice. Future multi-centre investigations with extended follow-up and comparative diagnostic assessments are necessary to validate these findings and explore their integration into routine care pathways.

## Conclusions

The findings of this study underscore the diagnostic and prognostic significance of serum tumour markers - CEA, CA50, CA19-9, CA72-4, and CYFRA21-1 - in colon cancer. These markers demonstrated clear associations with tumour positivity, disease staging, and the presence of recurrence or metastasis. Notably, the combined detection of all five markers significantly enhanced diagnostic accuracy compared to single-marker approaches, thereby offering greater utility for early screening and prognostic evaluation.

Despite these promising results, the study is limited by its relatively small sample size, which may affect the generalizability of the findings. Future research with larger cohorts is warranted to validate and extend these observations.

In summary, the combined detection of CEA, CA50, CA19-9, CA72-4, and CYFRA21-1 presents a valuable tool for the clinical diagnosis and management of colon cancer and merits broader implementation in clinical practice.

## Dodatak

### Acknowledgements

The authors extend their sincere appreciation to the staff of The Seventh Affiliated Hospital, Sun Yat-sen University, Shenzhen 518107, Guangdong Province, China, for their invaluable assistance throughout the study. We are also deeply grateful to the patients and their families for their participation and cooperation, without which this research would not have been possible.

### Funding

This research did not receive any specific grant from funding agencies in the public, commercial, or not-for-profit sectors.

### Data availability

The datasets generated and analysed during the current study are available from the corresponding author upon reasonable request.

### Authors' contribution

Ze Song conceptualised the study, collected clinical data, and contributed to manuscript preparation. Ze Song, Jun Tang, Min Wu and Zhi Li conducted data analysis and assisted in interpreting the results. Yingqiang Zhang provided study supervision and performed critical revisions for intellectual content.

All authors reviewed and approved the final version of the manuscript.

### Conflict of interest statement

All the authors declare that they have no conflict of interest in this work.
